# PKCθ/β and CYLD Are Antagonistic Partners in the NFκB and NFAT Transactivation Pathways in Primary Mouse CD3^+^ T Lymphocytes

**DOI:** 10.1371/journal.pone.0053709

**Published:** 2013-01-15

**Authors:** Nikolaus Thuille, Katarzyna Wachowicz, Natascha Hermann-Kleiter, Sandra Kaminski, Friedrich Fresser, Christina Lutz-Nicoladoni, Michael Leitges, Margot Thome, Ramin Massoumi, Gottfried Baier

**Affiliations:** 1 Department of Pharmacology and Genetics, Medical University of Innsbruck, Innsbruck, Austria; 2 The Biotechnology Centre of Oslo, Oslo, Norway; 3 Department of Biochemistry, University of Lausanne, Lausanne, Switzerland; 4 Department of Laboratory Medicine, Lund University, Malmö, Sweden; University of North Dakota, United States of America

## Abstract

In T cells PKCθ mediates the activation of critical signals downstream of TCR/CD28 stimulation. We investigated the molecular mechanisms by which PKCθ regulates NFκB transactivation by examining *PKCθ/β* single and double knockout mice and observed a redundant involvement of PKCθ and PKCβ in this signaling pathway. Mechanistically, we define a PKCθ-CYLD protein complex and an interaction between the positive PKCθ/β and the negative CYLD signaling pathways that both converge at the level of TAK1/IKK/I-κBα/NFκB and NFAT transactivation. In Jurkat leukemic T cells, CYLD is endoproteolytically processed in the initial minutes of stimulation by the paracaspase MALT1 in a PKC-dependent fashion, which is required for robust IL-2 transcription. However, in primary T cells, CYLD processing occurs with different kinetics and an altered dependence on PKC. The formation of a direct PKCθ/CYLD complex appears to regulate the short-term spatial distribution of CYLD, subsequently affecting NFκB and NFAT repressional activity of CYLD prior to its MALT1-dependent inactivation. Taken together, our study establishes CYLD as a new and critical PKCθ interactor in T cells and reveals that antagonistic PKCθ/β-CYLD crosstalk is crucial for the adjustment of immune thresholds in primary mouse CD3^+^ T cells.

## Introduction

The central role of PKCθ in signal transduction pathways during an adaptive immune response has extensively focused on the exact biochemical mechanisms of PKCθ function (reviewed in [1]–[3]). A recent study by Kong et al. identified the structural requirement in PKCθ for its localization to the immunological synapse as a prerequisite for activation of downstream signaling [4]. Several transcription factors essential for the T cell activation response (i.e. NFκB, AP1, and NFAT) are regulated by PKCθ [5], [6]. In vivo analysis of *PKCθ* ^−/−^ mice revealed the importance of PKCθ for Th2- [7] and Th17-mediated immune responses [8], [9] but not for host-protective antiviral responses [10]. Nevertheless, despite a profound understanding of the cellular role of PKCθ, little is known about its molecular function, specifically the effector proteins downstream of PKCθ during T cell activation.

Ubiquitylation and deubiquitylation are established posttranslational mechanisms for regulating immune responses, as well as the development and activation of immune cells. The tumor suppressor gene CYLD encodes an evolutionary conserved and ubiquitously expressed protein of approximately 120 kDa and was originally discovered as gene mutated in familial cylindromatosis, an autosomal dominant inherited disease characterized by the development of multiple benign skin tumors, principally on the head and neck [11]. Functionally it is a deubiquitylating enzyme (DUB) which removes mainly K63-linked polyubiquitin chains from several specific substrates, influencing in a negative way the activation status and/or spatial distribution of these target proteins in different signaling pathways. Numerous studies both in vitro and in vivo provided us with new insights in its established function as an important negative regulator of inflammatory responses, by counteracting the aberrant activation of NFκB signaling: *Cyld^−/−^* animals spontaneously develop intestinal inflammation and autoimmune symptoms due to the constitutive activation of the TAK1/IKK/IκBα axis [12], [13]; the study of Lim et al. described a CYLD dependent negative NFκB regulation during bacteria induced lung inflammation in mice via deubiquitylation of TRAF6 and TRAF7 [14]; moreover, the same scientific group showed that *Cyld* knockout mice are protected from Streptococcus pneumonia infection and lethality via a negative crosstalk with p38 MAPK [15]; a synergistic crosstalk between the E3 ligase Itch and CYLD for TAK1 inactivation and termination of tumor necrose factor dependent inflammatory signaling was recently described [16].

CYLD plays also an essential role in regulating T cell development and activation. *Cyld*-deficient mice show a delayed thymocyte development due to a constitutively K48-ubiquitylated and degraded LCK protein [17]. In addition, *Cyld*-deficient T cells are hyperresponsive to TCR/CD28 stimulation and CYLD has been firmly established as negative regulator of NFκB and JNK activation in response to antigen receptor activation in T cells [12], [13], [18].

In the current study, we defined physiologically redundant roles for the PKCθ and PKCβ isotypes in TCR/CD28-dependent NFκB and NFAT transactivation by examining *PKCθ/β* single and double knockout mouse lines. Additionally, we provide experimental evidence that a constitutive interaction of PKCθ with CYLD apparently leads to CYLD sequestration that affects the transactivation of the critical transcription factors NFκB and NFAT. Therefore, the results described here elucidate some aspects of PKCθ and PKCβ function during TCR activation and the processes that modulate CYLD function upstream of NFκB and NFAT activation in primary CD3^+^ T lymphocytes.

## Materials and Methods

### Mice

*PKCθ/β* knockout mice are viable, fertile and were generated by crossing *PKCθ* [5] and *PKCβ* [19] single knockout mice. The generation of *Cyld*-deficient mice was described previously [20]. All mice were on a C57Bl/6 background and housed (under SPF conditions) at the mouse facility of the Medical University of Innsbruck. All animal experiments were performed in accordance with the Austria “Tierversuchsgesetz” (BGBI. Nr. 501/1988 i.d.g.F.) and have been granted by the Bundesministerium für Bildung, Wissenschaft und Kultur (bm:bwk).

### Plasmids and Reagents

Strep-HA-tagged PKCθ and *Cyld* cDNAs (full-length and R324A mutant) were cloned into pEF-Neo. Vectors expressing full-length Flag-tagged wild-type CYLD or N- or C- terminally truncated forms of CYLD (encoding residues 1–212, 318–956 and 587–986 of CYLD) were described previously [21].

The pan-PKC low molecular weight inhibitor LMWI [22] was provided by NYCOMED GmbH, and the tetrapeptide inhibitor z-VRPR-fmk (MALT1 LMWI) was a gift from Dr. Margot Thome.

### Cell Culture and Transfections

Jurkat-TAg cells [23] (a kind gift from G.R. Crabtree, Stanford University, CA) were maintained in RPMI medium supplemented with 10% FCS (Life Technologies, Inc.) and antibiotics. Transient transfection of cells with 20 µg of plasmids encoding GFP, wild-type *Cyld* or a cleavage-resistant R324A *Cyld* mutant was performed by electroporation with a BTX-T820 Electro Square Porator (ITC, Biotech, Heidelberg, Germany) apparatus under predetermined optimal conditions: 2×10^7^ cells at 450 V/cm and five pulses of 99 ms.

HEK293T cells were cultured in Dulbecco’s Modified Eagle’s Medium supplemented with 10% FCS, 2 mM L-glutamine, and 100 µg/ml penicillin–streptomycin. HEK293T cells were transfected using MetafecteneTM transfection reagent according to the manufacturer instructions.

Primary human T cells were purified from PBMCs (isolated by standard Hypaque–Ficoll separation from whole blood samples) with the Pan T Cell Isolation Kit (Miltenyi Biotec) according to the manufacturer instructions.

Primary mouse CD3^+^ T cells were purified from pooled spleens and lymph nodes with mouse T cell enrichment columns (R&D Systems). T cell populations were typically 95% CD3^+^ as determined by staining and flow cytometry.

### Analysis of Proliferative Response and IL-2 Cytokine Production

For in vitro proliferation, 5×10^5^ T cells in 200 µl proliferation medium (RPMI supplemented with 10% FCS, 2 mM L-glutamine and 50 units/ml penicillin/streptomycin) were added in duplicate to 96-well plates precoated with anti-CD3 antibody (clone 2C11, 5 µg/ml) and soluble anti-CD28 (1 µg/ml; BD Bioscience) was added. For TCR-independent T cell stimulation, 10 ng/ml Phorbol 12,13-dibutyrate (PDBu) and 125 ng/ml of the calcium ionophore ionomycin were added to the media. Cells were harvested on filters after a 64 h stimulation period, pulsed with H^3^-thymidine (1 µCi/well) in the final 16 h and the incorporation of H^3^-thymidine was measured with a Matrix 96 direct β counter system.

For short time stimulation, cells were activated by the addition of anti-CD3 and anti-CD28 (or PDBu and ionomycin), both in soluble form. For crosslinking, anti-hamster IgG1 (clone HIG-632) was used.

IL-2 production in mouse CD3^+^ T cells after antibody stimulation was determined by BioPlex technology (BioRad Laboratories) from the supernatant.

### Western Blot Analysis

Cells were lysed in ice-cold lysis buffer [5 mM Na_3_VO_4_, 5 mM NaP2P, 5 mM NaF, 5 mM EDTA, 150 mM NaCl, 50 mM Tris (pH 7.3), 2% NP-40, 50 µg/ml aprotinin and leupeptin] and centrifuged at 15,000×*g* for 15 min at 4°C. Protein lysates were subjected to immunoblotting using antibodies against NFATc1 (Affinity Bioreagents), (p)S473 AKT, (p)ERK, ERK, (p)T183/Y185 JNK, JNK, p50, p65, (p)Y-783 PLCγ1, (p)T184/T187Tak1, Tak1 (all from Cell Signaling), PKCβ, PKCθ (both from BD Transduction Laboratories), actin, Cyld (E10), DNA polymerase and Fyn (all from Santa Cruz Biotechnology). The Cyld antibody recognizing the NH2-terminal region of Cyld was described previously [20].

### Co-immunoprecipitation Analysis

For co-immunoprecipitation, 1×10^7^ primary mouse T cells (or 1×10^6^ transiently transfected HEK293T cells) were lysed in 400 µl of immunoprecipitation buffer [5 mM Na_3_VO_4_, 5 mM NaP2P, 5 mM NaF, 5 mM EDTA, 150 mM NaCl, 1% NP-40, 50 mM Tris (pH 7.4), 50 mg/ml aprotinin and leupeptin]. Lysates were precleared for 1 h at 4°C. Immunoprecipitation was performed at 4°C overnight using 2 µg of the relevant antibodies. Thereafter, lysates were incubated with protein G Sepharose (Amersham-Pharmacia, Vienna) [for Streptactin IP, Streptactin Beads were used] for 1 h at 4°C, extensively washed in lysis buffer, resolved on an SDS–PAGE and immunostained for the relevant protein.

### Gel Mobility Shift Assays

Nuclear extracts were harvested from 1×10^7^ cells according to standard protocols. Briefly, purified CD3^+^ T cells were washed in PBS and resuspended in 10 mM HEPES (pH 7.9) 10 mM KCl, 0.1 mM EDTA, 0.1 mM EGTA, 1 mM DTT and protease inhibitors. Cells were incubated on ice for 15 min. NP-40 was added to a final concentration of 0.6%, cells were vortexed vigorously, and the mixture was centrifuged for 5 min. The nuclear pellets were washed twice and resuspended in 20 mM HEPES (pH7.9), 0.4 M NaCl, 1 mM EDTA, 1 mM EGTA, and 1 mM DTT and protease inhibitors, and the tube was rocked for 30 min at 4°C. After centrifugation for 10 min, the supernatant was collected. Extracted proteins (2 µg) were incubated in binding buffer with [32P]-labeled, double-stranded oligonucleotide probes (NFκB: 5′-GCC ATG GGG GGA TCC CCG AAG TCC-3′; NFAT: 5′-GCC CAA AGA GGA AAA TTT GTT TCA TAC AG-3′) (Nushift; Active Motif). In each reaction, 3×10^5^ c.p.m. of labeled probe was used, and the band shifts were resolved on 5% polyacrylamide gels. All experiments were performed at least three times with similar outcomes.

### Flow Cytometry

Single-cell suspensions from the spleen, lymph node and thymus were prepared and incubated for 30 min on ice in staining buffer (PBS containing 2% fetal calf serum and 0.2% NaN_3_) with FITC, PE or APC antibody conjugates. Surface marker expression was analyzed using a FACScan™ cytometer (Becton Dickinson & Co., Mountain View, CA) and CellQuestPro™ software according to standard protocols. Antibodies against murine CD3, CD4, and CD8 were obtained from Caltag Laboratories; CD19, CD69, CD44, and CD25 were obtained from BD PharMingen.

### Retroviral Transduction of Primary Mouse T cells

The packaging cell line platE was transfected with a pMX retroviral vector encoding an EGFP-*Cyld* fusion cDNA. Approximately 36 h later, supernatants were collected and used directly to infect 24 h to 48 h preactivated CD3^+^ cells using spin inoculation (1 h, 2000×*g*, 32°C), followed by a 5–6 h incubation period at 37°C. Infected cells were washed, resuspended in full supplemented medium and incubated for an additional 48 h to 72 h. From these cultures, GFP-expressing cells were analyzed using confocal microscopy to track the subcellular distribution of the protein of interest.

### Monitoring CYLD Localization Using Confocal Microscopy

CD3^+^ cells from wild type and *PKCθ/β* knockout mice that were transduced with a retrovirus expressing an EGFP-CYLD were not stimulated or PDBu- and ionomycin-stimulated, transferred to a polylysine-coated slide and fixed with 2% paraformaldehyde. After permeabilization (0.1% TritonX-100 in PBS) and a blocking step (5% goat serum in PBS), the cells were stained with Alexa595-CTB (for lipid raft staining) and TOPRO 3 (Nucleus) (Molecular Probes). Immunofluorescence was analyzed with a Zeiss LSM 510 confocal laser scanning microscope and Zeiss LSM software v3.2.

### Statistical Analysis

Differences between genotypes were analyzed using the unpaired Student’s t test.

## Results

### Overlapping Roles of PKCθ and PKCβ in NFκB and NFAT Transactivation Processes in Primary Mouse CD3^+^ T cells

**Figure 1:**
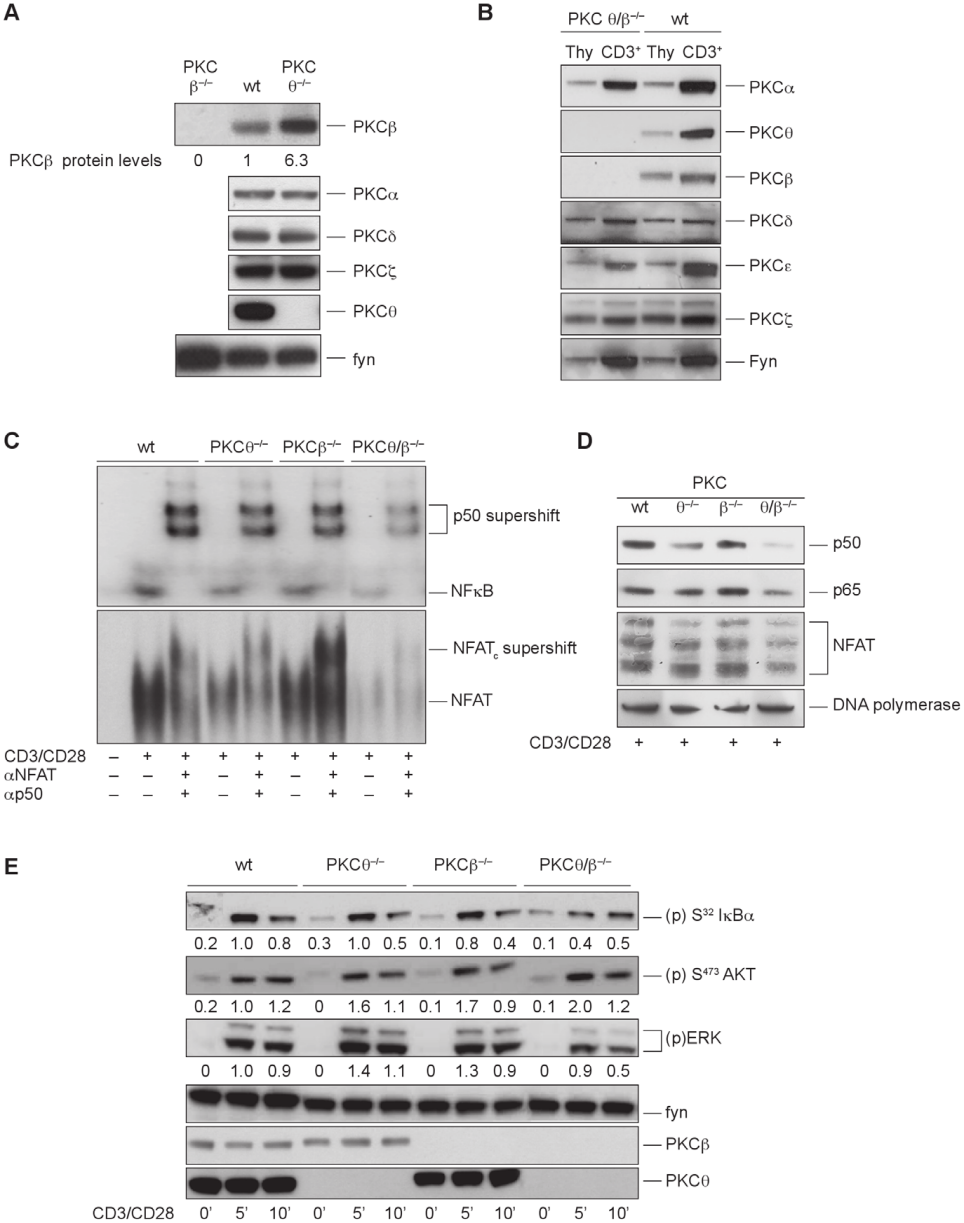
Overlapping roles of PKCθ and PKCβ in NFκB and NFAT transactivation processes in primary mouse CD3^+^ T cells. (A) PKCβ expression is upregulated in whole cell extracts of peripheral CD3^+^ T cells derived from *PKCθ*-deficient mice. (B) The PKC isoform expression profile in whole cell extracts of naive thymocytes (Thy) and peripheral CD3^+^ T cells derived from wild-type and *PKCθ/β^−/−^* mice. PKCθ/β inhibition leads to an increased NF-κB and NFAT transactivation defect in T cells. (C) The nuclear extracts of resting and stimulated (overnight) wild-type, *PKCθ^−/−^*, *PKCβ^−/−^* and *PKCθ/β^−/−^* CD3^+^ T cells were probed for DNA binding to radio-labeled probes containing NFκB and NFAT binding site sequences, as indicated. One representative experiment of three is shown. (D) Impaired nuclear import of p50, p65 and NFAT in activated *PKCθ/β*−-deficient T cells. Nuclear extracts of resting and stimulated (overnight) wild-type, *PKCθ^−/−^*, *PKCβ^−/−^* and *PKCθ/β^−/−^* CD3^+^ T cells were probed for p65, p50 and NFAT using immunoblot assays. DNA polymerase served as the loading control. One representative experiment of three is shown. (E) Effect of PKCθ/β inhibition on proximal phosphorylation events after a brief stimulation. Western blot analysis was performed with cytosolic extracts from wild-type, *PKCθ^−/−^*, *PKCβ^−/−^* and *PKCθ/β^−/−^* CD3^+^ T cells. CD3^+^ T cells were stimulated with anti-CD3/anti-CD28 and probed at different time points for the phosphorylation status of (p)S-32 IκBα, (p)S-473 AKT and (p)ERK1/2, as indicated. Fyn served as loading control. One representative experiment of three is shown. Protein phosphorylation levels were relatively quantitated by densitometric analysis. Numbers beneath bands indicate fold change compared to wt control after normalization to FYN.

Studies using targeted gene disruption defined a critical role for PKCθ in the activation of the IL-2 promoter in the NFκB and Ca^2+^/NFAT pathways [5], [6]. Surprisingly, the phenotypic characterization of *PKCθ*-deficient T cells revealed a strong upregulation of PKCβ protein levels in *PKCθ* single knockout T cells (Fig. 1A). To investigate potentially compensatory and overlapping roles of these two PKC family members in T cell activation processes, *PKCθ/β* double knockout mice were generated. These mice were viable, fertile and breed at normal Mendelian ratios. The null mutations for *PKCθ* and *PKCβ* were confirmed by PCR and immunoblotting of whole cell lysates from naive thymocytes and peripheral CD3^+^ T cells (Fig. 1B).

Flow cytometric analysis of thymocyte populations in *PKCθ/β^−/−^*double knockout mice revealed a slightly diminished percentage of CD3-, CD4- and CD8-positive cells, comparable to the *PKCθ* single knockout phenotype and in agreement with previous research [24], [25], which might indicate an involvement of PKCθ in the positive selection process during thymocyte development. Nevertheless, in the periphery, *PKCθ/β* ^−/−^ mice revealed no gross differences in the distribution of CD3-, CD4-, CD8-positive cells, leading to the conclusion that the concomitant loss of *PKCθ* and *PKCβ* did not additively affect T cell development (Table 1+ Table 2).

**Table 1:** Flow cytometric analyses of the cellularity of the thymus, spleen and lymph nodes from wild-type and *PKCθ/β−/−* mice.

Thymus	CD3^+^	CD19^+^	CD4^+^	CD8^+^	CD4^+^CD8^+^
wt	17,05±2,29	0,09±0,05	5,21±1,50	2,11±1,47	89,76±2,63
*PKCθ/β* ^−/−^	11,08±3,51	0,21±0,07	2,93±0,05	1,46±0,71	93,95±1,90
**Spleen**	**CD3^+^**	**CD19^+^**	**CD4^+^**	**CD8^+^**	**CD4^+^CD8^+^**
wt	22,84±8,57	66,86±16,63	17,16±4,59	9,89±2,52	0,90±0,98
*PKCθ/β* ^−/−^	26,59±3,79	63,37±5,24	14,97±2,33	13,70±1,92	0,88±0,72
**Lymphnodes**	**CD3^+^**	**CD19^+^**	**CD4^+^**	**CD8^+^**	**CD4^+^CD8^+^**
wt	53,31±0,56	37,92±2,28	36,49±0,30	20,81±0,34	1,51±0,73
*PKCθ/β* ^−/−^	53,14±1,31	39,84±0,37	31,74±1,91	24,50±3,27	1,09±0,37

Surface expression of CD3, CD4, CD8 and CD19 were measured by flow cytometry; the relative fluorescence intensities are indicated as a percentage of positive cells. The results shown are the mean±SE of three independent experiments.

**Table 2:** Absolute cell numbers of thymic populations from wild-type and *PKCθ/β−/−* mice.

	CD3^high^	CD4^+^	CD8^+^	CD4^+^CD8^+^
wt	27,1±1,3	8,2±1,6	3,3±2,0	143,8±16,9
*PKCθ/β* ^−/−^	17,2±2,5	4,7±0,9	2,2±0,7	150,6±29,6

Absolute cell numbers of thymic populations (x10^6^). The results shown are the mean±SE of three independent experiments.

Examination of the stimulation-dependent upregulation of CD25, CD69 and CD44 surface markers on CD4^+^ and CD8^+^ subsets revealed no gross differences in the total percentage of positive cells between the genotypes, but the total protein amount per cell, monitored by median fluorescence intensity, was strongly reduced in *PKCθ/β* ^−/−^ and to an intermediate extend in *PKC* singly-deficient T cells. These data might indicate a possible defect in the upregulation of both the IL-2 receptor chain alpha (CD25) and the activation marker CD69 in *PKC*-deficient T cells in both CD4^+^ and CD8^+^ T cells (Fig. S1).

In contrast to the relatively normal T-cell development observed, the T-cell response of peripheral T cells after TCR stimulation was affected by the single and simultaneous loss of *PKCθ* and *PKCβ*. The H^3^-thymidine uptake and IL-2 secretion response of *PKCθ/β-*deficient T cells stimulated with anti-CD3 and with or without anti-CD28-activated did not significantly exacerbate the defects already observed in the absence of *PKCθ* alone (Fig. S2A–C). To exclude the proliferative defects being caused by deregulated apoptosis, we analyzed the activation-induced cell death (AICD) of CD4^+^ and CD8^+^ T-cell blasts derived from wild-type and double knockout animals using CD3 engagement in vitro; in addition also the Fas ligand induced cell death was monitored, but no enhanced apoptotic responses of *PKCθ/β* ^−/−^ were detected (Fig. S3A–B).

However, analysis of the pathways leading to IL-2 transcription revealed additively reduced binding of NFκB and NFAT to DNA in *PKCθ/β* double-deficient CD3^+^ T cells after CD3/CD28 stimulation (Fig. 1C). Immunoblot analysis of nuclear extracts demonstrated that the weaker DNA binding of NFκB and NFAT was due to the reduced nuclear entry of two NFκB subunits, p50 and p65, and NFAT upon stimulation (Fig. 1D). Activation of NFκB involves the phosphorylation of I-κBα by IKKβ and its subsequent proteasomal degradation. Consistent with the additive affect on NFκB translocation, the double knockout showed a weaker I-κBα phosphorylation after stimulation with CD3/CD28. Also the activation of the Map kinase pathway was partially affected by PKCθ/β deficiency, visible through a reduced ERK phosphorylation, whereas the activation of Akt/PKB was normal (Fig. 1E).

### PKCθ and PKCβ Synergistically Regulate TAK1 and JNK Activation

**Figure 2:**
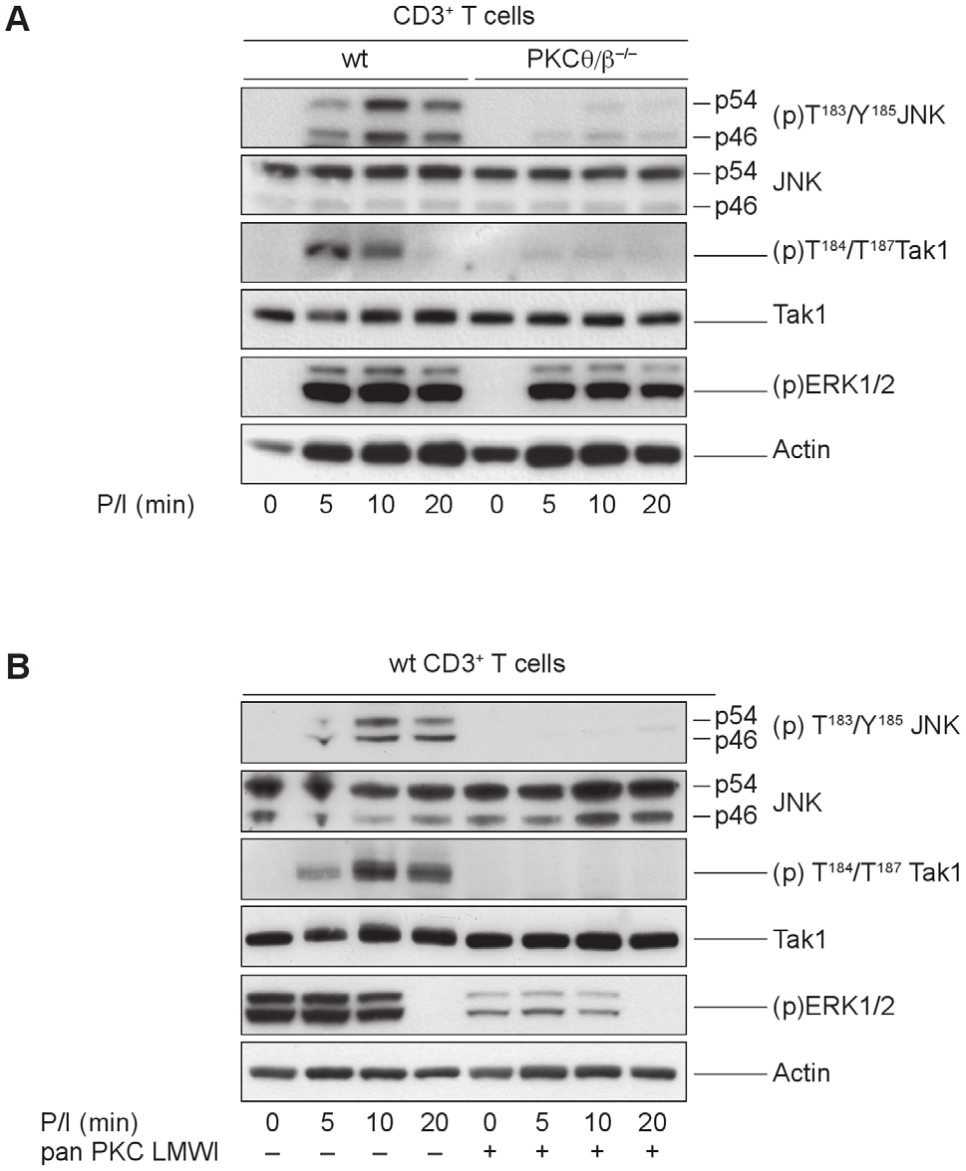
PKCθ and PKCβ synergistically regulate TAK1 and JNK activation. (A) Defective Tak1 and JNK activation in *PKCθ/β*-deficient CD3^+^ cells. Cytosolic extracts of PDBu- and ionomycin-stimulated wild-type and *PKCθ/β^−/−^* CD3^+^ T cells were probed for the phosphorylation status of TAK1, JNK and ERK1/2, as indicated. Actin served as loading control. One representative experiment of three is shown. (B) PKC enzymatic activity influences TAK1 and JNK activation status. The cytosolic extracts of PDBu- and ionomycin-stimulated, pan-PKC LMWI pretreated, or untreated control wild-type CD3^+^ T cells were probed for the phosphorylation status of Tak1, JNK and ERK1/2, as indicated. Actin served as loading control. One representative experiment of three is shown.

In agreement with the defective IKK/I-κBα axis, *PKCθ/β* ^−/−^ CD3^+^ cells revealed a drastic activation defect in TGF β activated kinase 1 (TAK1), which is known to be a key regulator of IKKβ signaling. The loss of both PKC isotypes appears to be required to abolish the signal, because TAK1 activation levels were similar between the wild-type and PKC single knockout T cells (not shown). Additionally, the JNK signal was attenuated by the targeted disruption of *PKCθ* and *PKCβ*, whereas ERK1/2 activation was only marginally affected (Fig. 2A). Similar outcomes were observed in PDBu- and ionomycin-stimulated primary mouse wild-type T cells pretreated with 500 nM of a PKC-specific low molecular weight inhibitor (PKC LMWI). The stronger effect of the pharmacological pan-PKC inhibitor on MAP kinase activation can be best explained by its established inhibition of additional PKC family members next to PKCθ and PKCβ (Fig. 2B).

### *Cyld* ^−/−^ T cells Show a Hyperactive Phenotype in NFκB and NFAT Transactivation Responses

Since the deubiquitinating enzyme CYLD has been shown to be a negative regulator of Tak1 [12] and JNK signaling [26], we investigated a possible link between CYLD and the PKCθ/β isotypes in NFκb and NFAT driven IL-2 upregulation.

**Figure 3:**
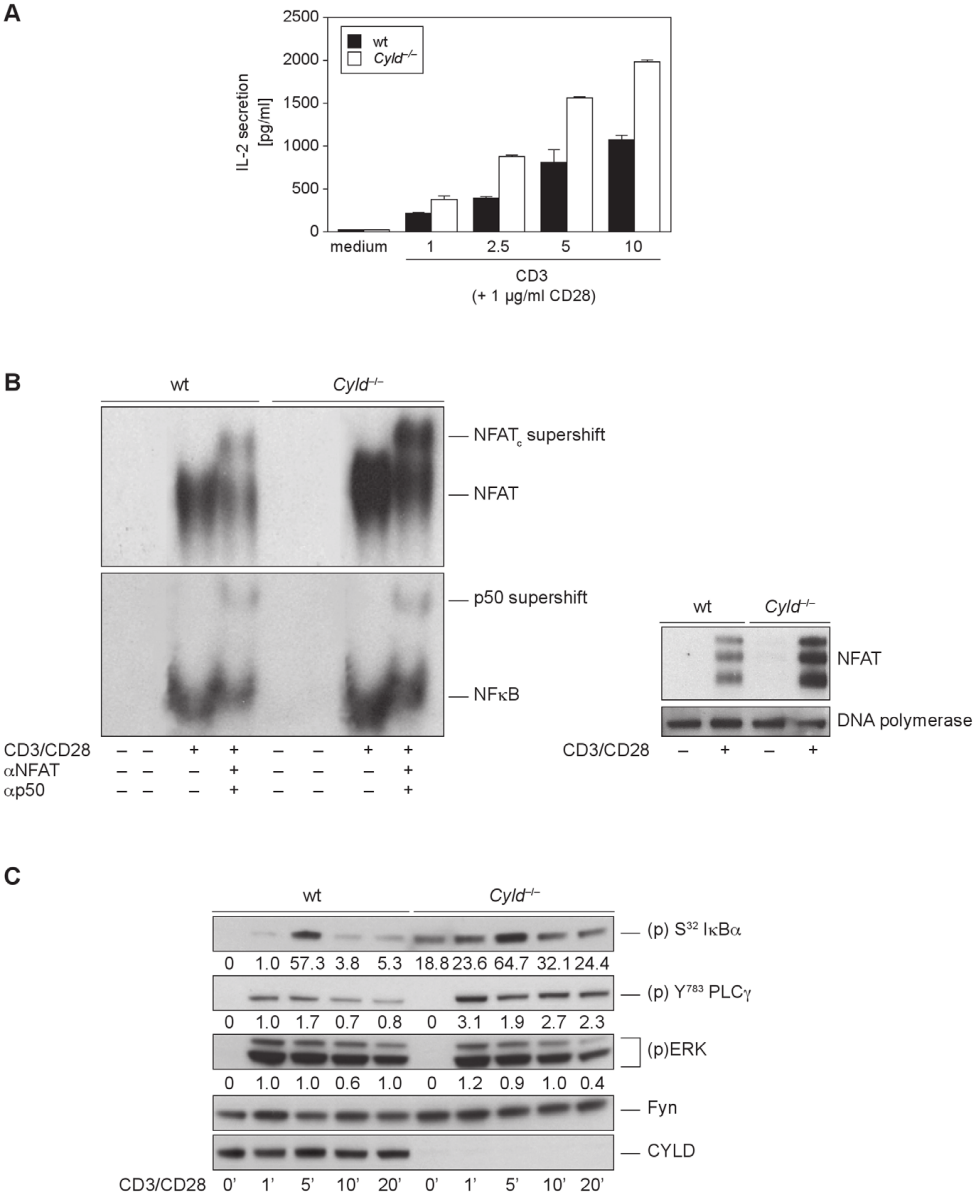
T cells from *Cyld^−/−^* mice exhibit hyper-responsiveness to TCR stimulation. (A) Naive wild-type and *Cyld* deficient CD3^+^ cells were stimulated overnight (16 h) with the indicated amount of plate-bound anti-CD3 and soluble anti-CD28. Cytokines in the supernatants were measured using Bioplex suspension array technology. (B) Nuclear extracts were isolated from unstimulated and CD3/CD28-stimulated CD3^+^ T cells from wild-type control and *Cyld* ^−/−^ mice. EMSA was performed to determine the activity of NFAT and NFκB. The same nuclear extracts were probed for NFAT and DNA polymerase, using immunoblot assays, the latter one served as the loading control. (C) Purified naive peripheral CD3^+^ T cells from wild-type and *Cyld* ^−/−^ mice were stimulated with anti-CD3 and anti-CD28 for the indicated time periods. Immunoblotting assays were performed using the indicated phospho-specific and pan-antibodies. Protein phosphorylation levels were relatively quantitated by densitometric analysis. Numbers beneath bands indicate fold change compared to wt control after normalization to FYN.

Despite of an observed thymocyte maturation defect, recent work on T cell signaling in *Cyld* ^−/−^ mice demonstrated hyper-responsiveness to TCR stimulation by constitutive activation of NFκB [12]. We confirmed these results, as we also observed that *Cyld* ^−/−^ T cells showed elevated activation-induced IL-2 responses (Fig. 3A). This hyper-responsive IL-2 secretion correlated with an increase of NFκB DNA binding to the IL-2 promoter in the nuclear fractions of stimulated *Cyld*-deficient T cells (Fig. 3B) and hyper-phosphorylated I-κBα levels in the cytosol compared to wild-type controls (Fig. 3C). Interestingly, and in accordance with a previous publication [27], we determined that CYLD also acts as a negative modulator of the NFAT pathway. The examination of NFAT transactivation using immunoblot and EMSA technology revealed increased nuclear translocation and subsequent binding of NFAT to DNA in *Cyld*-deficient cells (Fig. 3B). Our EMSA result was confirmed by the elevated activation status of phospholipase Cγ1, which has been identified as a key regulator of Ca2+/Calcineurin/NFAT signaling. However, ERK signaling was not affected by the loss of *Cyld* (Fig. 3C).

### Association of CYLD with PKCθ

**Figure 4:**
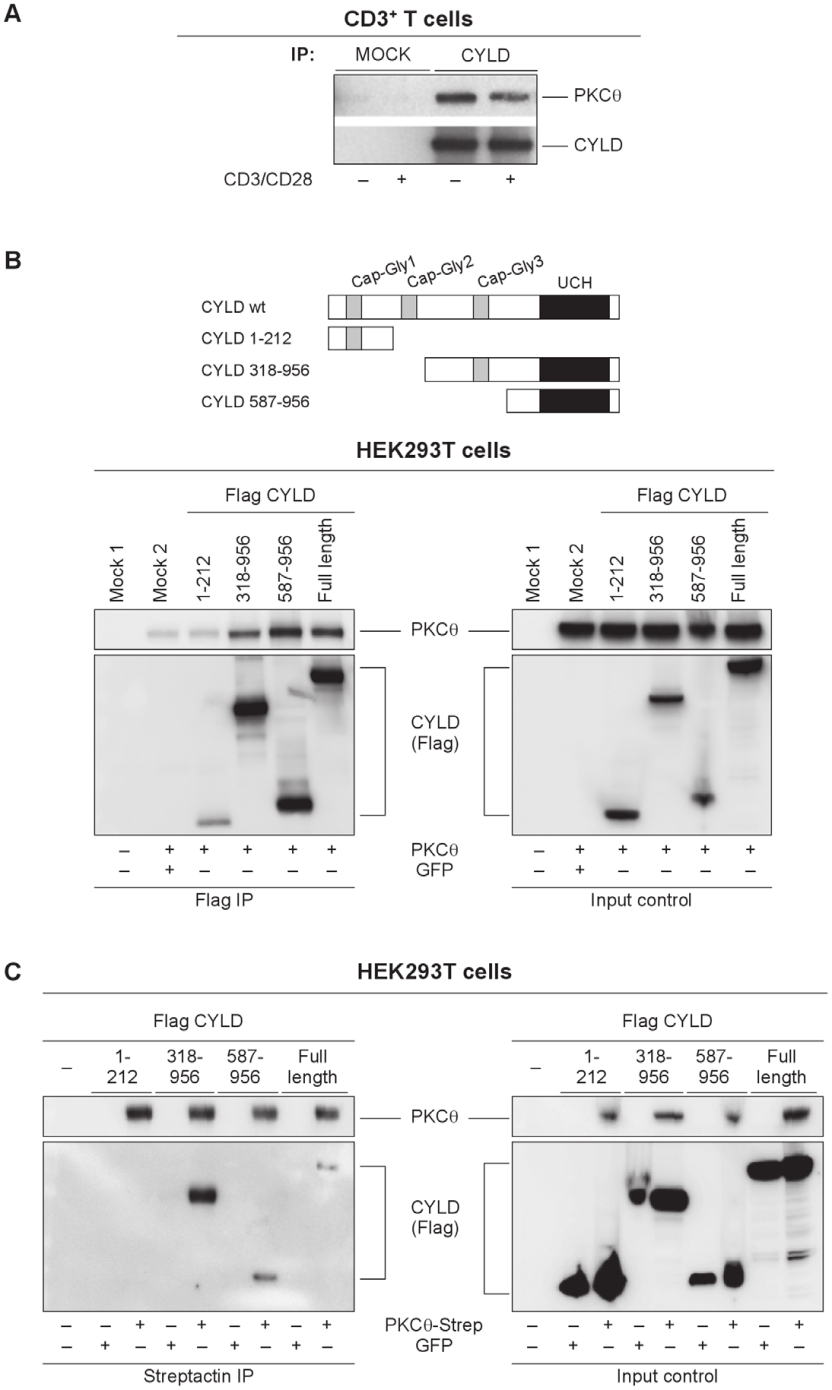
Association of CYLD with PKCθ. (A) CYLD directly interacts with PKCθ in primary mouse CD3^+^ cells. The complex is formed constitutively and is not affected by TCR activation. One representative experiment of three is shown. The C-terminal region of CYLD is important for interaction with PKCθ. (B) Co-immunoprecipitation of PKCθ using CYLD pulldown. Increased binding of PKCθ to the C-terminus of CYLD was shown in HEK293T cells transiently co-transfected with vectors encoding PKCθ (PEFneo) and a full-length Flag-tagged wild-type CYLD, N- or C-terminally truncated forms of CYLD (residues 1–212, 318–956 and 587–986 of CYLD). Untransfected and GFP-transfected controls were included. One representative experiment of three is shown. A schematic representation depicts the CAP-Gly and peptidase domains in wild-type and truncation mutants of CYLD. (C) Co-immunoprecipitation of CYLD using PKCθ pulldown. A strep-tagged full-length PKCθ construct was co-transfected with Flag-tagged CYLD constructs into HEK293T cells. As previously, the importance of the C-terminal region of CYLD for binding is shown. GFP controls for each CYLD construct were included. One representative experiment of three is shown.

Considering the reciprocal phenotypes of *Cyld*- and *PKCθ/β*-deficient T cells involving NFAT and NFκB transactivation we investigated a potential direct interaction between this enzymes. Interestingly and indeed, we identified a physical and functional PKCθ-CYLD interaction in the cytosol of primary T cells. The co-immunoprecipitation analysis of CYLD and the PKCθ isotype from cell extracts of unstimulated and CD3/CD28-activated peripheral CD3^+^ cells revealed that PKCθ and CYLD physically associate in a complex in resting conditions (Fig. 4A). Next, we mapped the PKCθ interaction domain in the CYLD protein by co-transfection of HEK293T cells with a vector encoding PKCθ and with vectors expressing full-length Flag-tagged wild-type or N- and C- terminally truncated forms of CYLD (encoding residues 1–212, 318–956 and 587–986 of CYLD). The CYLD pull down with a specific Flag antibody revealed increased binding of PKCθ to CYLD mutants containing the deubiquitinase domain (Fig. 4B). We observed identical results when the co-immunoprecipitation was performed to precipitate PKCθ. A strep-tagged PKCθ construct was co-transfected with the Flag-tagged CYLD constructs. A GFP control for each CYLD construct was included to identify unspecific binding to the Streptactin-beads. PKCθ precipitation confirmed that the C-terminal part of CYLD is necessary for complex formation between the two interacting protein (Fig. 4C).

### In Jurkat Cells, CYLD is Cleaved by MALT1 in a PKC-dependent Mechanism

**Figure 5:**
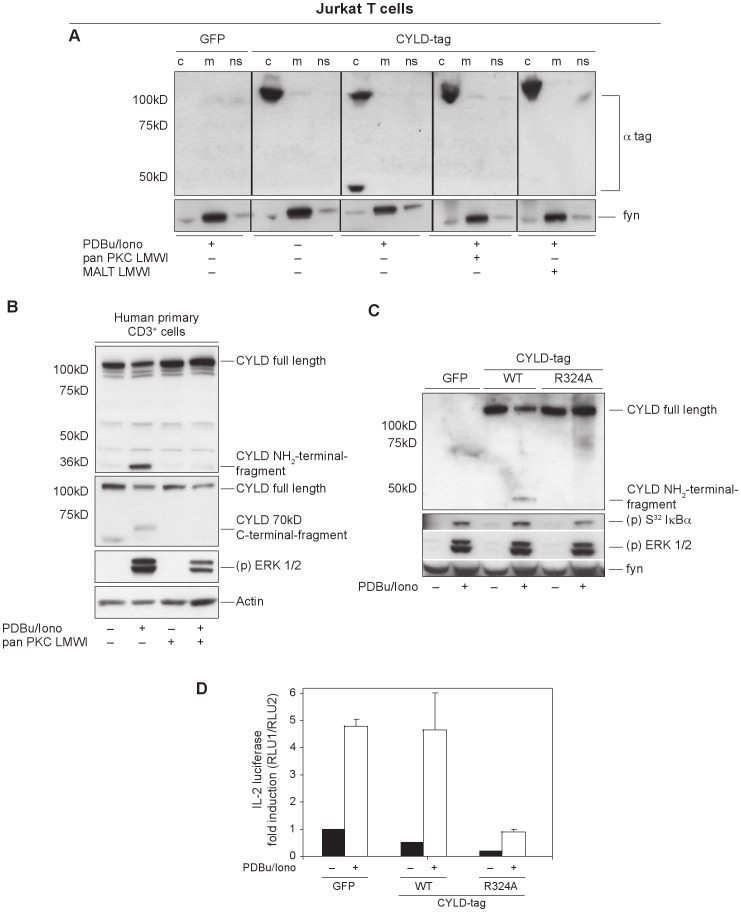
CYLD is endoproteolytically processed in the Jurkat cell line and in primary human T cells. (A) The essential role of both the catalytic activity of PKC and MALT1 for activation-dependent CYLD cleavage. Jurkat cells transfected with HA-tagged Cyld vectors were activated by PDBu and ionomycin in the presence of PKCθ and MALT1 pharmacological inhibitors. After 20 min, cells were lysed and fractionated into membrane (m), cytosolic (c) and nonsoluble (ns) fractions. CYLD and its clipping product were present in (c), inhibition of PKCθ and MALT1 activity blocks CYLD cleavage. (B) CYLD processing occurred also under endogenous conditions in stimulated primary human T cells isolated from whole blood samples; PKC dependency of this process was verified by pan-PKC LMWI treatment. The generation of a 40 kDa NH_2_-terminal and a 70 kDa C-terminal cleavage fragment was confirmed via the use of NH_2_- and C-terminus recognizing specific CYLD antibodies. (C) Mapping of the R234 cleavage site in CYLD using site-directed mutagenesis. The cleavage-resistant CYLD mutant R324A, when overexpressed in Jurkat cells, is not cleaved and leads to an increased suppression of NFκB-driven signals (as detected by the decreased phosphorylation of IκBα. (D) PKC- and MALT1-induced proteolysis at R234 appears critical for complete IL-2 promoter transactivation, shown by an IL-2 promoter luciferase assay.

When Jurkat cells overexpressing an N-terminally HA-tagged CYLD construct were stimulated for 30 min with PDBu and ionomycin, a CYLD fragment of approximately 40 kDa was detected using the anti-HA antibody. Because the administration of PDBu mimics TCR signaling by activating PKC family members, we wanted to identify a role for PKC in this cleavage event. Therefore, Jurkat cells were pretreated with the specific pan-PKC pharmacological inhibitor, which resulted in the disappearance of this fragment (Fig. 5A). This finding emphasized that the endoproteolytic cleavage of CYLD is PKC dependent. We also isolated primary T cells from human whole blood and analyzed the CYLD processing under endogenous conditions. In addition, human T cells were treated with the pan-PKC inhibitor to investigate the PKC dependency in this process. Comparable to the results with Jurkat cells, CYLD underwent a stimulation dependent processing also in primary human T cells, which could be blocked by PKC inhibition. The generation of a 40 kDa NH_2_-terminal and a 70 kDa C-terminal cleavage fragment was confirmed via the use of NH_2_- and C-terminus recognizing specific CYLD antibodies (Fig. 5B).

Because Coornaert et al. showed that the paracaspase MALT1 directly cleaved the deubiquitinating protein A20 to generate a fragment with a smaller molecular size in stimulated T cells [28], we asked if MALT1 was also responsible for the cleavage of CYLD by treating Jurkat cells with the tetrapeptide inhibitor z-VRPR-fmk, which has been shown to inhibit specifically the MALT1 protease activity [29]. As a result, cells treated with the MALT1 inhibitor showed a reduced CYLD cleavage after activation (Fig. 5A).

Based on the size and the molecular weight of the CYLD N-terminal 40 kDa proteolytic fragment, we identified the cleavable arginine residue at position 324 in the human CYLD protein and generated a *Cyld* mutant with alanine substituted for arginine at position 324 (*Cyld*-R324A). Next, we investigated if this mutant was cleavable when overexpressed in Jurkat cells or was resistant to proteolysis. Wild-type CYLD was processed after stimulation, whereas the mutant could no longer be cleaved (Fig. 5C). This led to permanent inhibition of the NFκB pathway by the inactivation resistant CYLD mutant and result in slightly diminished phosphorylation of I-κBα. The MAPK pathway was not affected by the expression of uncleavable CYLD.

Because NFκB is important for IL-2 upregulation in activated T cells, we tested the influence of the protease-resistant *Cyld* mutant on IL-2 transactivation using an IL-2-promoter-dependent luciferase assay. The diminished NFκB signal observed by immunoblot was correlated with impaired IL-2 transcription (Fig. 5D). This provides experimental evidence that CYLD processing, which leads to an inactivation of its repressor function within a positive feedback loop, is an important prerequisite for robust IL-2 activation. Consistent with our investigation of CYLD cleavage at arginine 324 in the Jurkat tumor cell line, Staal et al. independently published that TCR-induced JNK activation required CYLD proteolysis by MALT1 [18]. Nevertheless, our findings extend the function of CYLD cleavage to NFκB activation. To identify the physiological role of this candidate process, we examined primary CD3^+^ T cells derived from wild-type and knockout mice.

### PKC-dependence, Cleavage Site and Kinetics of CYLD Cleavage Differ in Primary Mouse T cells

**Figure 6:**
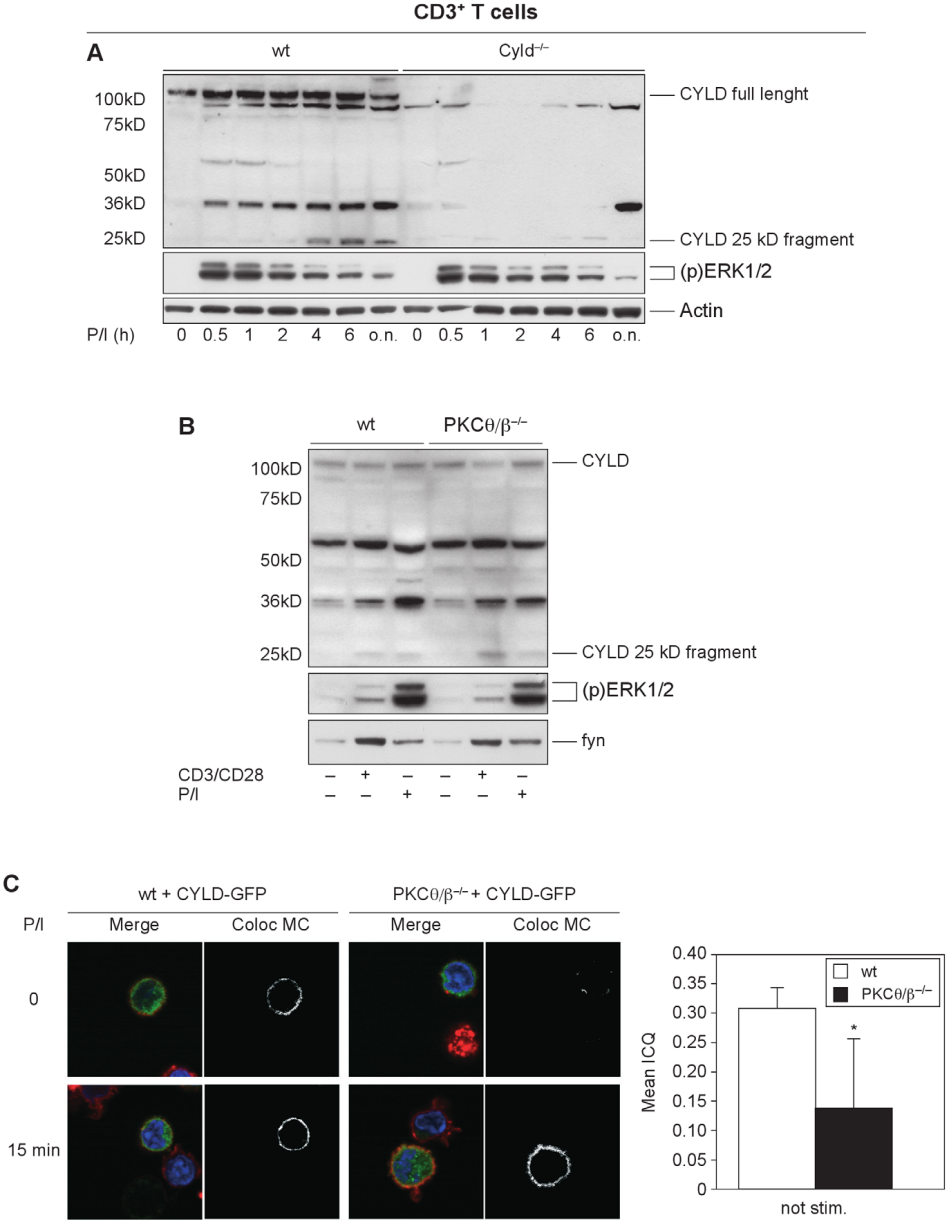
CYLD cleavage in primary mouse T cells shows a different kinetic. (A) CYLD cleavage in primary mouse cells has different kinetics. The activation of primary mouse T cells by CD3 with or without CD28 costimulation leads to the formation of an NH_2_-terminal CYLD fragment of approximately 25 kDa. In stark contrast to the rapid kinetics in Jurkat cells, the fragment was first detected after 4 h of stimulation. (B) Primary mouse T cells from *PKCθ/β^−/−^* mice showed normal stimulation-dependent CYLD cleavage, comparable to wild-type control mice. (C) Analysis of the subcellular distribution of a retrovirally introduced CYLD-GFP fusion mutant in unstimulated and stimulated wild-type and *PKCθ/β*-deficient T cells. (green): the co-localization with Cholera Toxin B stained lipid rafts (red) was monitored using confocal microscopy. Nuclei are stained in blue. Quantification of CYLD-lipid rafts co-localization is shown in the bars in the right panel and reveals a statistically significant decrease in CYLD translocation in unstimulated double knockout cells in comparison to wt control cells. *p<0.05; **p<0.01; ***p<0.001.

Stimulation induced a CYLD fragment not only in human cells but also in primary mouse T cells. Interestingly activation of primary mouse T cells by CD3 with or without CD28 costimulation generated a NH_2_-terminal CYLD fragment of approximately 25 kDa, smaller in size than the human fragment (Fig. 6A), implicating a different cleavage site in the mouse CYLD protein. Although we did not determine the exact cleavage site, arginine 235 was the best candidate for the cleavage site. Importantly, the fragment was first detectable after 4 h of stimulation, indicating different kinetics leading to CYLD inactivation in primary mouse cells. Unexpectedly, *PKCθ/β*-deficient T cells showed normal CYLD processing after stimulation, implicating additional protein kinases in this process (Fig. 6B). As a consequence, the activation defects of the TAK1/IKK axis in *PKCθ/β*-deficient T cells cannot solely be explained by CYLD inactivation.

Alternatively, the existence of a constitutive CYLD/PKCθ complex might suggest that PKCθ, which shows activation-dependent subcellular translocation, is important for removing CYLD from its NFκB-related targets and attenuates the negative regulatory function of CYLD, enabling feedback control of NFκB activation. To address this hypothesis, we analyzed the subcellular distribution of a retrovirally introduced mutant CYLD-GFP fusion protein in unstimulated and stimulated wild-type and *PKCθ/β*-deficient T cells with confocal microscopy. CD3^+^ T cells from both genotypes were retrovirally infected with a CYLD-GFP fusion construct, and the colocalization of CYLD-GFP with lipid rafts was monitored with Cholera Toxin B. Interestingly, CYLD colocalization with lipid rafts was strongly diminished in *PKCθ/β*-deficient T cells compared to control cells, suggesting PKC-dependent CYLD membrane shuttling. A statistically significant decrease in CYLD translocation in double knockout cells was detected in unstimulated cells, whereas after 15 min of PDBu and ionomycin stimulation, CYLD translocation to the membrane was observed in both wild-type and knockout cells (Fig. 6C).

## Discussion

Numerous studies emphasize PKCθ´s key role as a regulator of NFκB and Ca^2+^/NFAT signaling in T cells downstream of the TCR [5], [6], [30]–[32]. The PKCβ isotype is also expressed in T cells. However, *PKCβ*-deficient primary mouse T cells have a fairly normal activation response [33], although Volkov et al. established a major role for PKC*β* in LFA1-dependent T-cell locomotion [34], [35].

Our recent work defines a redundant role for both the novel PKCθ variant and the classical isotype PKCβ in the NFκB and NFAT signaling pathways. T cells isolated from *PKCθ/β*-deficient mice had a stronger impairment in NFκB and NFAT nuclear entry and DNA binding compared to CD3^+^ T cells from control and single knockout mice. Impaired TAK1 activation in double-deficient T lymphocytes is the best candidate for restricted IKK/IκBα signaling. Functional redundancy of PKCθ with other members of the PKC family in NFκB and/or NFAT activation has been shown in previous studies. For instance, stimulation-dependent colocalization of atypical PKCζ/ι with PKCθ in the lipid raft fraction of T lymphocytes leads to cooperation of these isotypes in modulating the NFκB signaling pathway [36]. The collaborative activity of PKCθ and PKCα in the NFAT pathway was examined in a *PKCα/θ* double knockout mouse strain. Compared to *PKCα* and *PKCθ* single-deficient T cells, double-deficient CD3^+^ cells showed additively reduced IL-2 secretion levels correlated with strongly impaired nuclear translocation and DNA binding of NFAT after stimulation. Of note, the *PKCα/θ* double knockout mice showed an impaired alloimmune response, leading to significantly prolonged allograft survival in heart transplantation experiments [37].

Similar to phosphorylation, K63 ubiquitination is a reversible process that influences protein activity, trafficking and signaling complex assembly. The removal of ubiquitin chains is mediated by a family of deubiquitinases, of which the cylindromatous gene product CYLD and the Tumor necrosis factor α-induced protein 3, also called A20, is currently receiving broad scientific attention. Both CYLD and A20 have been implicated as a modulator of the activity of NFκB-related molecules, such as NEMO (IKKγ), TRAF2 and TRAF6 [38]–[40]. Both enzymes overlap functionally by targeting a similar set of substrates, which was explained by the different expression pattern of A20 and CYLD. A20 function depends on its transcriptional upregulation, whereas CYLD is constitutively expressed, influencing the different time windows of NFκB activation differently. However, a constitutive expression and activity pattern requires a posttranslational regulatory mechanism to inactivate the repressor during signal-induced NFκB signaling.

The cleavage-dependent inactivation of a deubiquitinase as a posttranslational regulatory mechanism in activated T cells was first described by Coornaert et al. [28], in which A20 was defined as a MALT1 substrate, which upon antigen receptor engagement undergoes cleavage for functional NFκB signaling. In our study, we showed that PKCθ and CYLD are constitutively bound in a physical complex in the cytosol of primary mouse CD3^+^ cells. Direct crosstalk between CYLD and a PKC family member has not been described to date; therefore, we aimed to elucidate the biological relevance of this protein-protein interaction in T-cell signaling by examining genetic knockout mouse models in combination with selective pharmacological inhibitors. The reciprocal phenotypes of T-cell signaling pathways in *Cyld^−/−^* and *PKCθ/β^−/−^* mice prompted us to analyze the activity of key molecules linked to NFκB and NFAT transactivation to uncover a regulatory mechanism to address the modulation of TAK1 activity In agreement with Koga et al. [27], who demonstrated negative regulation of NFAT activity by CYLD via the TAK1/MKK3/6/p38α/β axis, our experimental data clearly attest to CYLD involvement in NFAT activity modulation downstream of TCR signaling.

Our results show that PKC, particularly the PKCθ/β isotypes, can influence CYLD repressive activity in different ways. In the human Jurkat leukemic T-cell line, PKC enzymatic activity was important for rapid MALT1-dependent CYLD processing, which is required for TCR-linked NFκB transactivation and leads to functional IL-2 induction. The requirement for MALT1-mediated CYLD cleavage for intact JNK signaling downstream of the TCR has been previously described [18]. Thus, proteolytic inactivation of CYLD affected IL-2 transactivation via the JNK/AP1 pathway; here, we provide experimental evidence that NFκB activity is also specifically dependent on CYLD cleavage, subsequently modulating IL-2 signals. Of note, we independently confirmed arginine 324 as the CYLD cleavage site. Recently, caspase 8 has been shown to cleave CYLD at aspartate 215 in Jurkat cells following TNFα stimulation, generating a pro-survival signal to save the cells from necrotic cell death [41]. Additionally, phosphorylation of CYLD was found to downregulate CYLD activity: transient phosphorylation by IKK in a serine cluster just upstream of the TRAF2 binding site, attenuates DUB function [42]. However, NFκB itself can regulate CYLD expression in a negative feedback loop [43].

In primary T cells isolated from *PKCθ/β* deficient mice, CYLD was processed to the same extent as in wild-type control cells. Additionally, the kinetics of CYLD cleavage was different in mouse T cells compared to Jurkat cells, starting approximately 4 hours after stimulation, later then the rapid response through TAK1 activation. The different requirement for PKC and the altered kinetics in the mouse system led to the analysis of the stimulation-dependent spatial and temporal organization of the PKCθ/CYLD complex using immunofluorescence microscopy. Interestingly, we found decreased CYLD lipid raft localization in *PKCθ/β-*deficient T cells under resting conditions, likely affecting activation-induced signaling.

### Conclusion

We observed a direct functional connection between the positive PKCθ/β and the negative CYLD signaling pathways that fine-tune TCR/CD28-induced signaling responses. Our findings suggest the following scenario: PKCθ/β are the essential kinases in a physiological signaling cascade that is necessary to counteract CYLD-mediated repression of NFκB and NFAT transactivation. This direct and physical antagonistic crosstalk between the PKC-derived signals and the CYLD-derived signals might represent one mechanism of how antigen-receptor-dependent fine-tuning of the amplitude of T lymphocyte activation is processed.

## Supporting Information

Figure S1 **Effect of PKCθ/β deficiency on CD25, CD44, and CD69 surface expression.** T cells were stimulated for 16 h by CD3/CD28 ligation and the surface expression of CD25, CD44, and CD69 for CD4^+^ and CD8^+^ subsets were measured by flow cytometry. The relative fluorescence intensities are indicated as the median fluorescence intensity. The results shown are the mean±SE of three independent experiments.
(TIF)

Figure S2 **Proliferative and cytokine secretion responses of** ***PKCθ/β*** **CD3^+^ T cells.** (A, B) Proliferative responses of *PKCθ/β* and PKCθ-deficient CD3^+^ T cells were analyzed in comparison to wild-type littermate controls. After incubation using different stimulatory conditions (antibodies or BALB/C splenocytes), cells were analyzed using standard procedures for thymidine incorporation. (C) IL-2 cytokine secretion by knockout CD3^+^ T cells was analyzed in comparison to wild-type littermate controls. After stimulation with anti-CD3 with or without soluble anti-CD28, supernatants were analyzed for IL-2 concentration using Bioplex suspension array technology. One representative experiment of three is shown.
(TIF)

Figure S3 **Activation-induced cell death (AICD) of CD4^+^ and CD8^+^ T cell blasts derived from double knockout animals was not increased compared to cells from single knockout littermates.** (A, B) AICD was induced by different concentrations of anti-CD3 for 8 hours. The results shown are the means of three independent experiments.
(TIF)

## References

[1] Hayashi K, Altman A (2007) Protein kinase C theta (PKCtheta): a key player in T cell life and death. Pharmacol Res 55: 537–544. 17544292 10.1016/j.phrs.2007.04.009 PMC2045646

[2] Marsland BJ, Kopf M (2008) T-cell fate and function: PKC-theta and beyond. Trends Immunol 29: 179–185. 18328786 10.1016/j.it.2008.01.005

[3] Sedwick CE, Altman A (2004) Perspectives on PKCtheta in T cell activation. Mol Immunol 41: 675–686. 15220003 10.1016/j.molimm.2004.01.007

[4] Kong KF, Yokosuka T, Canonigo-Balancio AJ, Isakov N, Saito T, et al (2011) A motif in the V3 domain of the kinase PKC-theta determines its localization in the immunological synapse and functions in T cells via association with CD28. Nat Immunol 12: 1105–1112. 21964608 10.1038/ni.2120 PMC3197934

[5] Pfeifhofer C, Kofler K, Gruber T, Tabrizi NG, Lutz C, et al (2003) Protein kinase C theta affects Ca2+ mobilization and NFAT cell activation in primary mouse T cells. J Exp Med 197: 1525–1535. 12782715 10.1084/jem.20020234 PMC2193906

[6] Sun Z, Arendt CW, Ellmeier W, Schaeffer EM, Sunshine MJ, et al (2000) PKC-theta is required for TCR-induced NF-kappaB activation in mature but not immature T lymphocytes. Nature 404: 402–407. 10746729 10.1038/35006090

[7] Marsland BJ, Soos TJ, Spath G, Littman DR, Kopf M (2004) Protein kinase C theta is critical for the development of in vivo T helper (Th)2 cell but not Th1 cell responses. J Exp Med 200: 181–189. 15263025 10.1084/jem.20032229 PMC2212016

[8] Salek-Ardakani S, So T, Halteman BS, Altman A, Croft M (2005) Protein kinase Ctheta controls Th1 cells in experimental autoimmune encephalomyelitis. J Immunol 175: 7635–7641. 16301673 10.4049/jimmunol.175.11.7635

[9] Tan SL, Zhao J, Bi C, Chen XC, Hepburn DL, et al (2006) Resistance to experimental autoimmune encephalomyelitis and impaired IL-17 production in protein kinase C theta-deficient mice. J Immunol 176: 2872–2879. 16493044 10.4049/jimmunol.176.5.2872

[10] Giannoni F, Lyon AB, Wareing MD, Dias PB, Sarawar SR (2005) Protein kinase C theta is not essential for T-cell-mediated clearance of murine gammaherpesvirus 68. J Virol 79: 6808–6813. 15890920 10.1128/JVI.79.11.6808-6813.2005 PMC1112139

[11] Bignell GR, Warren W, Seal S, Takahashi M, Rapley E, et al (2000) Identification of the familial cylindromatosis tumour-suppressor gene. Nat Genet 25: 160–165. 10835629 10.1038/76006

[12] Reiley WW, Jin W, Lee AJ, Wright A, Wu X, et al (2007) Deubiquitinating enzyme CYLD negatively regulates the ubiquitin-dependent kinase Tak1 and prevents abnormal T cell responses. J Exp Med 204: 1475–1485. 17548520 10.1084/jem.20062694 PMC2118606

[13] Zhang J, Stirling B, Temmerman ST, Ma CA, Fuss IJ, et al (2006) Impaired regulation of NF-kappaB and increased susceptibility to colitis-associated tumorigenesis in CYLD-deficient mice. J Clin Invest 116: 3042–3049. 17053834 10.1172/JCI28746 PMC1616194

[14] Lim JH, Jono H, Koga T, Woo CH, Ishinaga H, et al (2007) Tumor suppressor CYLD acts as a negative regulator for non-typeable Haemophilus influenza-induced inflammation in the middle ear and lung of mice. PLoS One 2: e1032. 17925880 10.1371/journal.pone.0001032 PMC2001183

[15] Lim JH, Stirling B, Derry J, Koga T, Jono H, et al (2007) Tumor suppressor CYLD regulates acute lung injury in lethal Streptococcus pneumoniae infections. Immunity 27: 349–360. 17723219 10.1016/j.immuni.2007.07.011

[16] Ahmed N, Zeng M, Sinha I, Polin L, Wei WZ, et al (2011) The E3 ligase Itch and deubiquitinase Cyld act together to regulate Tak1 and inflammation. Nat Immunol 12: 1176–1183. 22057290 10.1038/ni.2157 PMC3219826

[17] Reiley WW, Zhang M, Jin W, Losiewicz M, Donohue KB, et al (2006) Regulation of T cell development by the deubiquitinating enzyme CYLD. Nat Immunol 7: 411–417. 16501569 10.1038/ni1315

[18] Staal J, Driege Y, Bekaert T, Demeyer A, Muyllaert D, et al (2011) T-cell receptor-induced JNK activation requires proteolytic inactivation of CYLD by MALT1. EMBO J 30: 1742–1752. 21448133 10.1038/emboj.2011.85 PMC3101995

[19] Leitges M, Schmedt C, Guinamard R, Davoust J, Schaal S, et al (1996) Immunodeficiency in protein kinase cbeta-deficient mice. Science 273: 788–791. 8670417 10.1126/science.273.5276.788

[20] Massoumi R, Chmielarska K, Hennecke K, Pfeifer A, Fassler R (2006) Cyld inhibits tumor cell proliferation by blocking Bcl-3-dependent NF-kappaB signaling. Cell 125: 665–677. 16713561 10.1016/j.cell.2006.03.041

[21] Wickstrom SA, Masoumi KC, Khochbin S, Fassler R, Massoumi R (2010) CYLD negatively regulates cell-cycle progression by inactivating HDAC6 and increasing the levels of acetylated tubulin. EMBO J 29: 131–144. 19893491 10.1038/emboj.2009.317 PMC2775896

[22] Hermann-Kleiter N, Thuille N, Pfeifhofer C, Gruber T, Schafer M, et al (2006) PKCtheta and PKA are antagonistic partners in the NF-AT transactivation pathway of primary mouse CD3+ T lymphocytes. Blood 107: 4841–4848. 16514061 10.1182/blood-2005-10-4044

[23] Northrop JP, Ullman KS, Crabtree GR (1993) Characterization of the nuclear and cytoplasmic components of the lymphoid-specific nuclear factor of activated T cells (NF-AT) complex. J Biol Chem 268: 2917–2923. 8428966

[24] Gruber T, Pfeifhofer-Obermair C, Baier G (2010) PKCtheta is necessary for efficient activation of NFkappaB, NFAT, and AP-1 during positive selection of thymocytes. Immunol Lett 132: 6–11. 20433868 10.1016/j.imlet.2010.04.008 PMC2937209

[25] Morley SC, Weber KS, Kao H, Allen PM (2008) Protein kinase C-theta is required for efficient positive selection. J Immunol 181: 4696–4708. 18802072 10.4049/jimmunol.181.7.4696 PMC2645034

[26] Reiley W, Zhang M, Sun SC (2004) Negative regulation of JNK signaling by the tumor suppressor CYLD. J Biol Chem 279: 55161–55167. 15496400 10.1074/jbc.M411049200

[27] Koga T, Lim JH, Jono H, Ha UH, Xu H, et al (2008) Tumor suppressor cylindromatosis acts as a negative regulator for Streptococcus pneumoniae-induced NFAT signaling. J Biol Chem 283: 12546–12554. 18332137 10.1074/jbc.M710518200 PMC2335367

[28] Coornaert B, Baens M, Heyninck K, Bekaert T, Haegman M, et al (2008) T cell antigen receptor stimulation induces MALT1 paracaspase-mediated cleavage of the NF-kappaB inhibitor A20. Nat Immunol 9: 263–271. 18223652 10.1038/ni1561

[29] Rebeaud F, Hailfinger S, Posevitz-Fejfar A, Tapernoux M, Moser R, et al (2008) The proteolytic activity of the paracaspase MALT1 is key in T cell activation. Nat Immunol 9: 272–281. 18264101 10.1038/ni1568

[30] Kingeter LM, Schaefer BC (2008) Loss of protein kinase C theta, Bcl10, or Malt1 selectively impairs proliferation and NF-kappa B activation in the CD4+ T cell subset. J Immunol 181: 6244–6254. 18941215 10.4049/jimmunol.181.9.6244 PMC2630173

[31] Matsumoto R, Wang D, Blonska M, Li H, Kobayashi M, et al (2005) Phosphorylation of CARMA1 plays a critical role in T Cell receptor-mediated NF-kappaB activation. Immunity 23: 575–585. 16356856 10.1016/j.immuni.2005.10.007

[32] Wang D, Matsumoto R, You Y, Che T, Lin XY, et al (2004) CD3/CD28 costimulation-induced NF-kappaB activation is mediated by recruitment of protein kinase C-theta, Bcl10, and IkappaB kinase beta to the immunological synapse through CARMA1. Mol Cell Biol 24: 164–171. 14673152 10.1128/MCB.24.1.164-171.2003 PMC303359

[33] Thuille N, Gruber T, Bock G, Leitges M, Baier G (2004) Protein kinase C beta is dispensable for TCR-signaling. Mol Immunol 41: 385–390. 15163535 10.1016/j.molimm.2004.03.007

[34] Volkov Y, Long A, Kelleher D (1998) Inside the crawling T cell: leukocyte function-associated antigen-1 cross-linking is associated with microtubule-directed translocation of protein kinase C isoenzymes beta(I) and delta. J Immunol 161: 6487–6495. 9862672

[35] Volkov Y, Long A, McGrath S, Ni Eidhin D, Kelleher D (2001) Crucial importance of PKC-beta(I) in LFA-1-mediated locomotion of activated T cells. Nat Immunol 2: 508–514. 11376337 10.1038/88700

[36] Gruber T, Fresser F, Jenny M, Uberall F, Leitges M, et al (2008) PKCtheta cooperates with atypical PKCzeta and PKCiota in NF-kappaB transactivation of T lymphocytes. Mol Immunol 45: 117–126. 17588663 10.1016/j.molimm.2007.05.003

[37] Gruber T, Hermann-Kleiter N, Pfeifhofer-Obermair C, Lutz-Nicoladoni C, Thuille N, et al (2009) PKC theta cooperates with PKC alpha in alloimmune responses of T cells in vivo. Mol Immunol 46: 2071–2079. 19356803 10.1016/j.molimm.2009.02.030

[38] Brummelkamp TR, Nijman SM, Dirac AM, Bernards R (2003) Loss of the cylindromatosis tumour suppressor inhibits apoptosis by activating NF-kappaB. Nature 424: 797–801. 12917690 10.1038/nature01811

[39] Kovalenko A, Chable-Bessia C, Cantarella G, Israel A, Wallach D, et al (2003) The tumour suppressor CYLD negatively regulates NF-kappaB signalling by deubiquitination. Nature 424: 801–805. 12917691 10.1038/nature01802

[40] Trompouki E, Hatzivassiliou E, Tsichritzis T, Farmer H, Ashworth A, et al (2003) CYLD is a deubiquitinating enzyme that negatively regulates NF-kappaB activation by TNFR family members. Nature 424: 793–796. 12917689 10.1038/nature01803

[41] O'Donnell MA, Perez-Jimenez E, Oberst A, Ng A, Massoumi R, et al (2011) Caspase 8 inhibits programmed necrosis by processing CYLD. Nat Cell Biol 13: 1437–1442. 22037414 10.1038/ncb2362 PMC3229661

[42] Reiley W, Zhang M, Wu X, Granger E, Sun SC (2005) Regulation of the deubiquitinating enzyme CYLD by IkappaB kinase gamma-dependent phosphorylation. Mol Cell Biol 25: 3886–3895. 15870263 10.1128/MCB.25.10.3886-3895.2005 PMC1087725

[43] Jono H, Lim JH, Chen LF, Xu H, Trompouki E, et al (2004) NF-kappaB is essential for induction of CYLD, the negative regulator of NF-kappaB: evidence for a novel inducible autoregulatory feedback pathway. J Biol Chem 279: 36171–36174. 15226292 10.1074/jbc.M406638200

