# Supplementary figures and images for: PKCθ/β and CYLD Are Antagonistic Partners in the NFκB and NFAT Transactivation Pathways in Primary Mouse CD3+ T Lymphocytes
Source: PLoS One. 2013 Jan 15;8(1):e53709. doi: 10.1371/journal.pone.0053709 (PMC3546006; doi:10.1371/journal.pone.0053709)

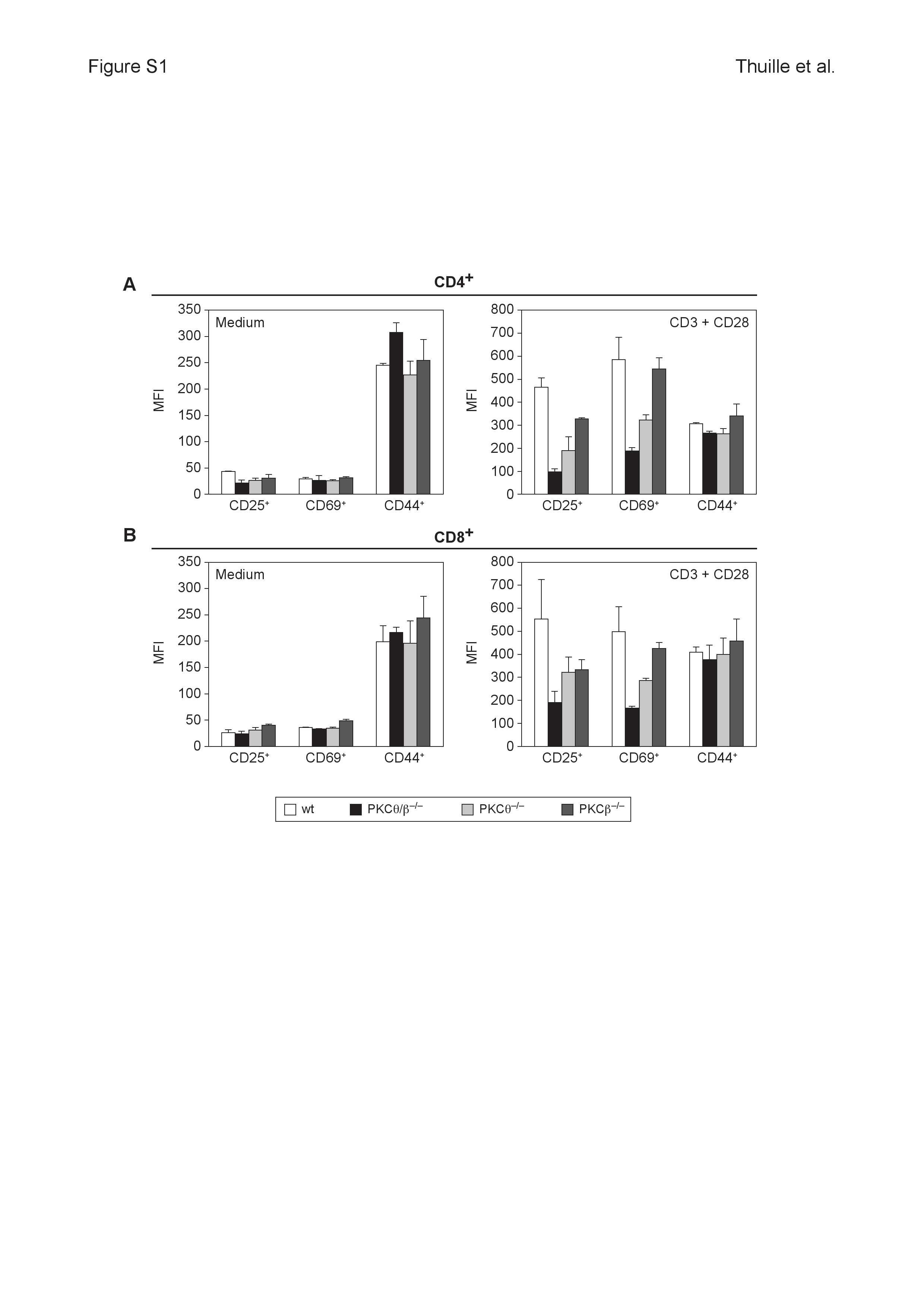

Supplement: Figure S1 — Effect of PKCθ/β deficiency on CD25, CD44, and CD69 surface expression. T cells were stimulated for 16 h by CD3/CD28 ligation and the surface expression of CD25, CD44, and CD69 for CD4+ and CD8+ subsets were measured by flow cytometry. The relative fluorescence intensities are indicated as the median fluorescence intensity. The results shown are the mean±SE of three independent experiments. (TIF) [file pone.0053709.s001.tif]

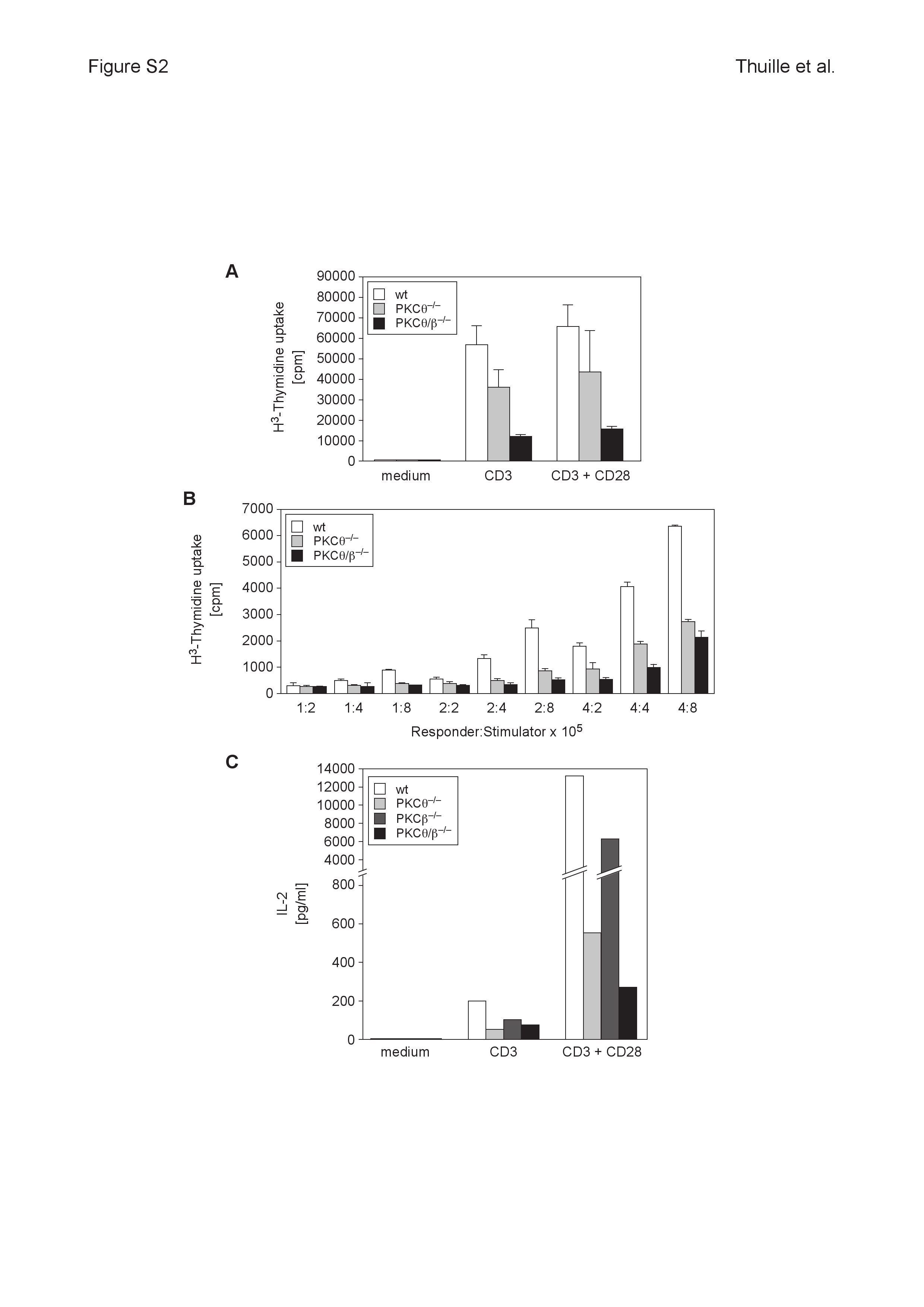

Supplement: Figure S2 — Proliferative and cytokine secretion responses of PKCθ/β CD3+ T cells. (A, B) Proliferative responses of PKCθ/β and PKCθ-deficient CD3+ T cells were analyzed in comparison to wild-type littermate controls. After incubation using different stimulatory conditions (antibodies or BALB/C splenocytes), cells were analyzed using standard procedures for thymidine incorporation. (C) IL-2 cytokine secretion by knockout CD3+ T cells was analyzed in comparison to wild-type littermate controls. After stimulation with anti-CD3 with or without soluble anti-CD28, supernatants were analyzed for IL-2 concentration using Bioplex suspension array technology. One representative experiment of three is shown. (TIF) [file pone.0053709.s002.tif]

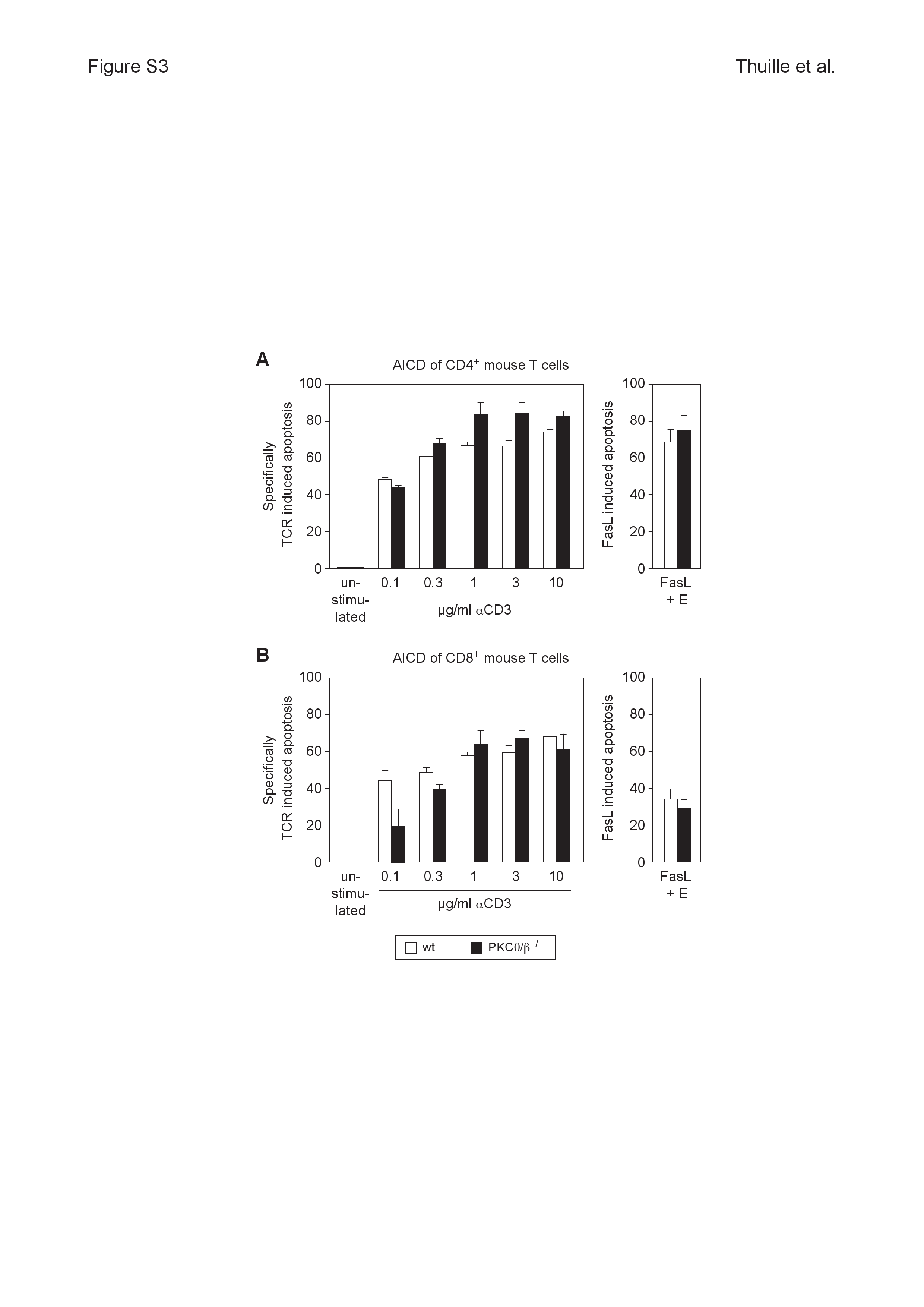

Supplement: Figure S3 — Activation-induced cell death (AICD) of CD4+ and CD8+ T cell blasts derived from double knockout animals was not increased compared to cells from single knockout littermates. (A, B) AICD was induced by different concentrations of anti-CD3 for 8 hours. The results shown are the means of three independent experiments. (TIF) [file pone.0053709.s003.tif]
